# Exploring the Help-Seeking Experience of Concerned Persons: Findings From an Elder Abuse UK Helpline

**DOI:** 10.1093/geroni/igaa057.2146

**Published:** 2020-12-16

**Authors:** Silvia Fraga Dominguez, Jennifer Storey, Emily Glorney

**Affiliations:** 1 Royal Holloway, University of London, Egham, United Kingdom; 2 University of Kent, Canterbury, England, United Kingdom; 3 Royal Holloway, University of London, Egham, England, United Kingdom

## Abstract

Despite their potential role in elder abuse cases, knowledge about concerned persons outside of North America is scarce. This paper will discuss findings from a study focusing on concerned persons in the UK, by addressing their profile, the impact of helping, and several variables relating to help-seeking. Researchers used secondary data from a charity’s helpline, encompassing a year of recorded cases (N = 1623). Concerned persons (n = 1352) were often related to the victim (80%) and/or perpetrator (59%). In 43% of cases, they reported impact as a result of their awareness of the situation or supporting the victim. This impact was thematically analysed and ranged widely in terms of severity, from slight worry to being subjected to the perpetrator’s homicide threats, and it often affected the person’s mental health or financial situation. Concerned persons reported substantial barriers to action relating to the access to and responses from formal services.

